# A One Health Computational Framework for Identifying PA Endonuclease Inhibitors Against Contemporary H5N1 Avian Influenza

**DOI:** 10.3390/vetsci13040385

**Published:** 2026-04-16

**Authors:** Manos C. Vlasiou

**Affiliations:** Department of Veterinary Medicine, School of Veterinary Medicine, University of Nicosia, 2414 Nicosia, Cyprus; vlasiou.m@unic.ac.cy

**Keywords:** H5N1 avian influenza, PA endonuclease, molecular docking, molecular dynamics, drug repurposing, One Health, computational toxicology, antiviral discovery

## Abstract

Highly pathogenic avian influenza (HPAI) H5N1 clade 2.3.4.4b continues to circulate globally across wild birds, poultry, and an expanding range of mammalian hosts, highlighting the need for antiviral strategies that address the animal–environment–human interface. The influenza A polymerase acidic (PA) endonuclease, a key enzyme in viral transcription, represents a conserved antiviral target across host species. In this study, we present a computational prioritization framework integrating homology modeling, molecular docking, molecular dynamics simulations, and physicochemical filtering to identify candidate PA endonuclease inhibitors relevant to a One Health context. Homology models of contemporary H5N1 clade 2.3.4.4b PA sequences were constructed based on the crystallographic template 6FS8 and used for cross-host docking against a targeted ligand library. Docking analysis identified baloxavir, a reference inhibitor, and entecavir, a nucleoside analog, as compounds of interest, with entecavir demonstrating favorable binding behavior, particularly in the poultry-associated model. Molecular dynamics simulations of the poultry PA–entecavir complex indicated stable interaction over 170 ns, supported by low structural deviation and favorable binding free energy (ΔG ≈ −85 kJ/mol). Physicochemical profiling suggested that entecavir possesses properties such as high polarity and predicted aqueous solubility, which were considered within the translational filtering step of this computational workflow. However, these properties do not establish antiviral efficacy or practical suitability for field use. The study provides a structured framework for integrating cross-host structural analysis with basic translational considerations, supporting the identification of candidate compounds for further biochemical and virological evaluation within the context of H5N1 control.

## Simple Summary

Avian influenza H5N1 continues to spread globally among birds and has increasingly infected mammals, raising concerns for animal health, food security, and potential human transmission. Current antiviral strategies are limited, particularly for use in poultry systems where practical constraints such as residue formation and environmental safety must be considered. In this study, we developed a computational framework to identify antiviral candidates targeting the influenza PA endonuclease, a key enzyme required for viral replication. Using structural modeling, molecular docking, and molecular dynamics simulations, we evaluated candidate compounds across both avian and mammalian viral variants. Entecavir emerged as a computationally prioritized candidate in the poultry-associated model; however, the present study does not demonstrate antiviral activity, inhibitory potency, or practical applicability. This work provides a strategy for prioritizing antiviral compounds that are not only biologically active but also suitable for real-world use in poultry production, supporting a One Health approach to controlling avian influenza.

## 1. Introduction

Highly pathogenic avian influenza (HPAI) H5N1 remains one of the most consequential viral threats at the animal–human interface [1,2,3]. Since 2021, the expansion of clade 2.3.4.4b across Europe, Asia, Africa, and the Americas has led to unprecedented levels of viral persistence in wild birds and repeated incursions into commercial poultry flocks [4,5]. The same period has seen an increase in spillover events into mammals, including farmed mink, domestic cats, marine mammals, and, most recently, dairy cattle, raising renewed concerns for zoonotic potential and pandemic preparedness [6,7,8]. Although vaccination strategies are expanding, effective antiviral countermeasures for poultry do not exist, and environmental contamination in barns and processing facilities remains a major driver of transmission during outbreaks [9,10,11].

The PA endonuclease, a component of the influenza polymerase complex, is essential for viral transcription by mediating cap-snatching [12,13,14]. Because this catalytic domain is structurally conserved across avian and mammalian isolates, it has emerged as a high-value antiviral target [15,16,17]. Baloxavir marboxil, a PA endonuclease inhibitor approved for human use, demonstrates the tractability of the target but also highlights limitations: reduced efficacy against specific variants, potential for resistance, and, importantly, unsuitability for use in poultry due to regulatory, residue, and pharmacokinetic constraints [18,19]. As a result, there is significant interest in identifying novel small molecules that inhibit the PA endonuclease but may exhibit (i) greater chemical simplicity, (ii) improved solubility for water-based delivery, (iii) reduced persistence in animal tissues, or (iv) compatibility with environmental or surface applications in poultry production systems [20,21,22]. Recent computational studies have evaluated potential PA endonuclease inhibitors using docking and molecular dynamics simulations [23,24,25]. However, existing work has three significant gaps:

1. Most studies focus on older H5N1 or H1N1 isolates, not the contemporary clade 2.3.4.4b lineage responsible for the current global spread [26,27].
2. To the best of our knowledge, no study incorporates cross-host structural comparisons (poultry vs. mammalian variants) to ensure antiviral candidates are robust across One Health transmission interfaces.
3. Critically, no computational pipeline has evaluated antiviral candidates in the context of poultry/agrochemical feasibility, including solubility, environmental safety, residue risk, and suitability for in-barn, water, aerosol, or surface delivery systems.

These unaddressed gaps limit the translational relevance of existing computational research. In a real outbreak, an antiviral compound must be biophysically potent but also chemically appropriate for field deployment in poultry operations, whether as a therapeutic adjunct, an environmental antiviral in barns and equipment, or as a tool to reduce viral shedding and thereby lower occupational exposure risk for workers [28,29,30,31].

**Figure 1:**
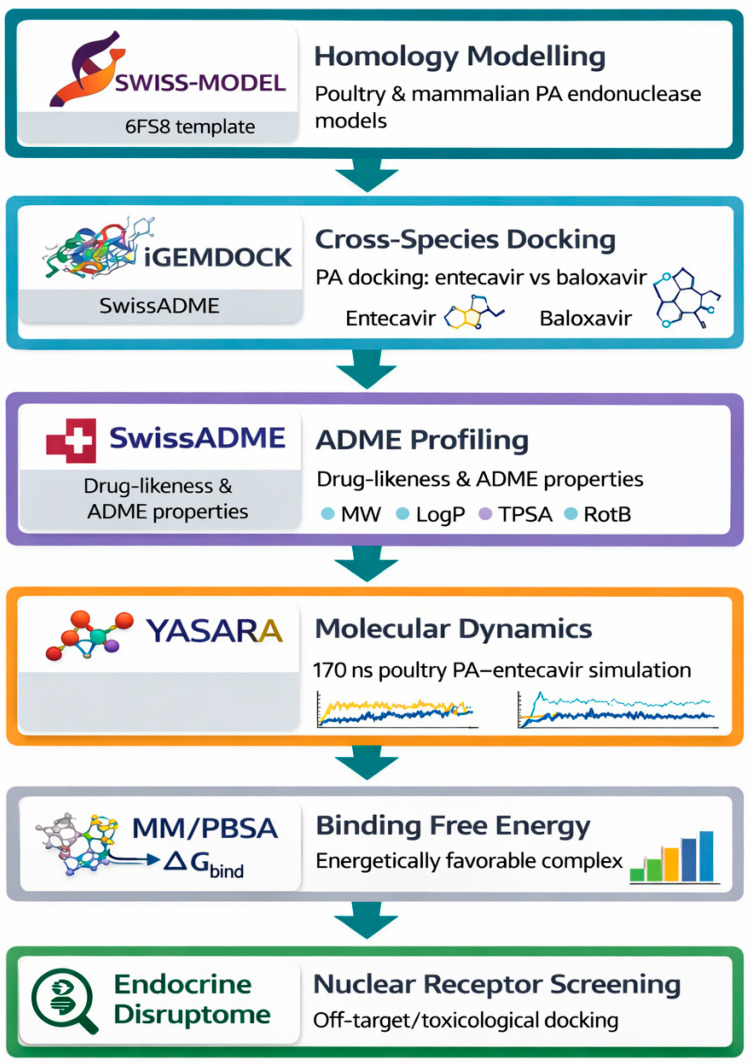
Overview of the computational pipeline used to evaluate entecavir versus baloxavir against influenza A PA endonuclease. The workflow integrates template-based homology modeling (SWISS-MODEL; template 6FS8), cross-species docking (iGEMDOCK), ADME profiling (SwissADME), molecular dynamics simulation (YASARA; 170 ns for poultry PA–entecavir), MM/PBSA binding free energy estimation, and nuclear receptor screening (Endocrine Disruptome).

In this study, we introduce a novel, integrative computational workflow designed explicitly for a One Health antiviral strategy. We (i) model the PA endonuclease from contemporary poultry-associated and mammalian-associated H5N1 2.3.4.4b variants, (ii) screen a library of repurposed antivirals and hydrophilic, low-residue scaffolds using metal-aware docking, (iii) evaluate stability using molecular dynamics and MM/GBSA free-energy calculations, (iv) assess resistance robustness, and (v) apply a poultry environment suitability filter incorporating solubility, toxicity, and predicted environmental behavior. Finally, we classify candidate inhibitors by translational use potential: poultry-directed antivirals, cross-host inhibitors, worker protection candidates, and environmental antiviral agents (Figure 1).

Together, these components form a computational and translational pipeline aimed at identifying antiviral compounds aligned with the practical realities of controlling H5N1 across poultry, environment, and human exposure pathways. This approach supports both veterinary public health and pandemic preparedness by targeting a conserved viral function while grounding compound selection in real-world field constraints.

## 2. Materials and Methods

### 2.1. PA Sequence Retrieval and Alignment

PA sequences from H5N1 clade 2.3.4.4b (2021–2025) were retrieved from GenBank/GISAID. Sequences representing wild birds, poultry, and mammalian spillover hosts were aligned (MAFFT v7). Variants with complete coding regions and no ambiguous nucleotides were selected. Representative poultry and mammalian PA variants were chosen for homology modeling.

### 2.2. Structural Template Identification

High-resolution PA endonuclease structures were screened in the Protein Data Bank. PDB 6FS8 (1.9 Å, inhibitor-bound, with intact Mn^2+^ ions) was selected as the primary template due to structural completeness and well-resolved catalytic geometry. Additional structures (4E5G, 5E6X, 3EBE, 3HW6) were used as secondary references to cross-validate the alignment and active-site geometry.

### 2.3. Homology Modeling

Homology models of the influenza A polymerase acidic (PA) endonuclease domain were constructed to represent both poultry-associated and mammalian-associated H5N1 clade 2.3.4.4b variants. Target amino acid sequences were retrieved from the NCBI database based on the following criteria: (i) recent isolates (post-2020), (ii) full-length PA segment coverage, and (iii) clear host annotation (avian or mammalian). Structural modeling was performed using the SWISS-MODEL server, employing the crystallographic structure of influenza A PA endonuclease (PDB ID: 6FS8) as the template. This structure was selected for its high resolution and relevance as a reference for PA endonuclease inhibitor binding, including complexes with baloxavir. Sequence alignment between target sequences and the template was automatically generated and manually inspected to ensure that catalytically relevant residues were correctly aligned. Particular attention was paid to the conservation of the metal-coordinating active-site residues (His41, Glu80, Asp108, and Glu119), which are critical for endonuclease function and inhibitor binding.

Model quality was evaluated using multiple complementary metrics provided by SWISS-MODEL, including GMQE (Global Model Quality Estimation), reflecting expected model reliability; QMEANDisCo global score, assessing agreement with experimentally derived structures; and local quality estimates, particularly within the active site region.

The final selected models exhibited high reliability (GMQE ≈ 0.90–0.95; QMEANDisCo ≈ 0.85–0.92), indicating strong structural agreement with the template. Structural superposition of the generated models with the template confirmed preservation of the catalytic core architecture and overall fold. Only models retaining correct metal coordination and without steric clashes were accepted for docking [32].

### 2.4. Active-Site Definition and Protein Preparation

The catalytic site was defined using conserved PA residues (H41, E80, D108, E119) and supporting residues (K34, T20, W88, R124, T123, K134). A 10–12 Å radius around metal ions defined the docking search space. All structures were prepared using Auto Dock Tools. Protonation states were assigned at pH 7.4. Metal ions were retained. Crystallographic waters were removed unless contributing bridging interactions. Minimization was performed to remove clashes.

### 2.5. Ligand Library Preparation

Three ligand sets were examined: 1. Reference PA inhibitors. 2. Hydrophilic, poultry/environment-compatible chemotypes. 3. FDA-approved antivirals or chelators. Ligands were protonated at pH 7.4, minimized via MMFF94, and converted into 3D conformers (Table 1, Table 2 and Table 3).

### 2.6. Docking

Docking was performed using iGEMDOCK v2.1. Each ligand was docked into poultry, mammalian, and crystal PA structures in triplicate. Poses were selected based on predicted affinity, correct metal-chelating geometry, catalytic residue interactions, and cross-host pose conservation (RMSD < 2 Å). The docking protocol was validated by redocking baloxavir into the crystallographic PA endonuclease structure (PDB 6FS8). The predicted pose reproduced the crystallographic orientation with RMSD < 2 Å, confirming the reliability of the docking settings.

### 2.7. ADMET and Poultry/Environmental Suitability

SwissADME, pkCSM, and ProTox-II were used for ADMET prediction. Chemical suitability criteria included: high solubility, low lipophilicity, low predicted avian/human toxicity, low environmental persistence, and minimal bioaccumulation risk. Compounds were classified into Tier 1 (poultry/environment-compatible), Tier 2 (uncertain), or Tier 3 (excluded).

### 2.8. Molecular Dynamics and Binding Energy

MD simulations were performed only for the top poultry-model complex: poultry PA–entecavir. The complex was simulated under NPT conditions (170 ns, YASARA AMBER 96 force field). Trajectories were analyzed for RMSD, RMSF, total potential energy, and secondary structure. The binding free energy was calculated using gmx_MMPBSA. MD simulations were performed using YASARA Dynamics with the AMBER96 force field. YASARA trajectories were exported in a compatible format and processed using gmx_MMPBSA. The system was solvated in a cubic water box using the TIP3P water model with periodic boundary conditions. Na^+^/Cl^−^ ions were added to neutralize the system. Temperature was maintained at 298 K using a Berendsen thermostat and pressure at 1 atm using a barostat. A timestep of 2 fs was used, and trajectories were recorded every 10 ps (Table 4).

## 3. Results

### 3.1. Homology Modeling and Structural Readiness of PA Endonuclease Targets

**Figure 2:**
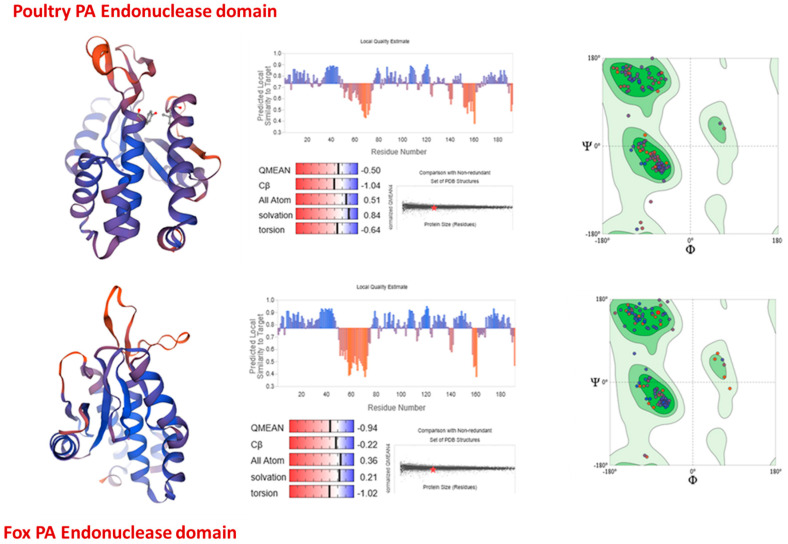
Structural alignment of SWISS-MODEL homology models of poultry- and mammalian-associated PA endonuclease domains (residues 1–193) against the crystallographic template (6FS8). The conserved catalytic pocket region is preserved in both models, supporting their suitability for structure-based docking.

Homology models of the poultry-associated and mammalian-associated PA endonuclease domains were successfully generated using the crystal structure PDB 6FS8 as the primary template. The modeling focused on residues 1–193, corresponding to the catalytically active endonuclease domain. Structural alignment of the two models with the template confirmed preservation of the characteristic α/β fold of the PA endonuclease catalytic domain and conservation of the metal-binding catalytic residues H41, E80, D108, and E119 (Figure 2).

The resulting models displayed high structural similarity to the template and retained the catalytic cavity geometry required for metal-dependent endonuclease activity. Visual inspection confirmed correct positioning of the catalytic pocket and absence of steric clashes that could interfere with ligand docking. These structural features indicate that the homology models were suitable for structure-based virtual screening and for cross-host comparison of ligand-binding behavior.

### 3.2. Cross-Species Docking Performance and Interaction Residues

Docking energies indicated robust engagement of both ligands with PA endonuclease across crystal and model targets. Baloxavir produced strong docking energies on 6FS8 (−101.6 to −101.7 across repeated runs), and similarly favorable scores on poultry and mammalian models (−97.5 to −97.7). Entecavir showed the strongest docking in the poultry model (−100.6), while remaining favorable in the mammalian model (−95.0) and in 6FS8 (−83). Residue-level interaction analysis revealed that baloxavir consistently engages catalytic pocket residues in both model and crystal targets. For the mammalian model, recurrent interactions were observed with Glu80, Arg84, Tyr24, Phe105, and Leu106. For the poultry model, baloxavir binding also involved residues in the catalytic cavity, including His41, Glu80, Asp108, Tyr24, and Arg84.

The redocking procedure successfully reproduced the crystallographic binding orientation of baloxavir within the PA endonuclease active site, yielding an RMSD of <2.0 Å. In addition, baloxavir showed highly consistent docking energies across replicate runs in the crystal structure, with scores of −101.7, −101.7, and −101.6, further supporting the internal consistency of the docking settings. These findings indicate that the docking protocol was able to reproduce the experimentally observed binding mode of a validated PA endonuclease inhibitor and was, therefore, suitable for subsequent screening of candidate compounds.

Entecavir docking produced target-specific residue signatures. In 6FS8, interactions included Arg85, Leu107, Glu120, Lys135, and Tyr106. In the mammalian model, entecavir interacted frequently with His41, Glu80, Arg82, Leu106, Pro107, and Asp108. In the poultry model, the top entecavir pose engaged residues, including His52, Ser60, Lys113, Ala159, Asp160, Thr162, Leu163, and Asp164, supporting stable positioning within the modeled pocket region (Table 5). Docking and interaction analysis support a consistent catalytic pocket binding hypothesis and cross-species feasibility, with baloxavir serving as a strong positive control and entecavir showing particularly favorable docking in the poultry model. Visual superposition confirmed close overlap between crystallographic and redocked poses within the catalytic pocket.

### 3.3. Interaction Profiles Within the Catalytic Pocket

Residue-level interaction analysis revealed that both ligands bind within the catalytic cavity of the PA endonuclease, engaging residues known to contribute to substrate recognition and metal-dependent catalysis. Baloxavir displayed highly consistent interaction profiles across the crystal and homology targets. In the mammalian PA model, the ligand formed recurrent interactions with Glu80, Arg84, Tyr24, Phe105, and Leu106, residues located within the catalytic pocket and surrounding hydrophobic cavity. In the poultry PA model, additional interactions were observed with His41 and Asp108, two residues directly involved in the metal-coordinating catalytic center. These interactions support the established mechanism of PA inhibition, in which the ligand chelates catalytic metal ions and stabilizes its binding through hydrogen bonding and hydrophobic contacts.

**Figure 3:**
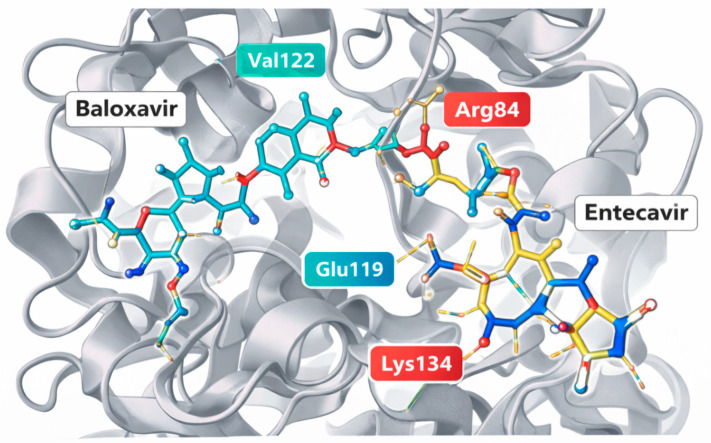
Representative iGEMDOCK docking pose of baloxavir and entecavir within the PA endonuclease active site. Key interacting residues identified by iGEMDOCK interaction analysis are highlighted. The pose illustrates binding within the catalytic cavity.

Entecavir demonstrated a somewhat different interaction pattern. Within the crystal structure, interactions were observed with Arg85, Leu107, Glu120, Lys135, and Tyr106, suggesting accommodation within the catalytic pocket but in a different orientation than in baloxavir. In the mammalian model, entecavir established contacts with His41, Glu80, Arg82, Leu106, Pro107, and Asp108, placing the ligand in proximity to the catalytic metal-binding residues. Interestingly, in the poultry PA model, the ligand engaged residues, including His52, Ser60, Lys113, Ala159, Asp160, Thr162, Leu163, and Asp164, suggesting stabilization through interactions extending beyond the immediate catalytic residues into the surrounding pocket region (Figure 3). This expanded interaction network may explain the improved docking score observed for entecavir in the poultry model.

### 3.4. Molecular Dynamics Stability of the Poultry PA–Entecavir Complex

**Figure 4:**
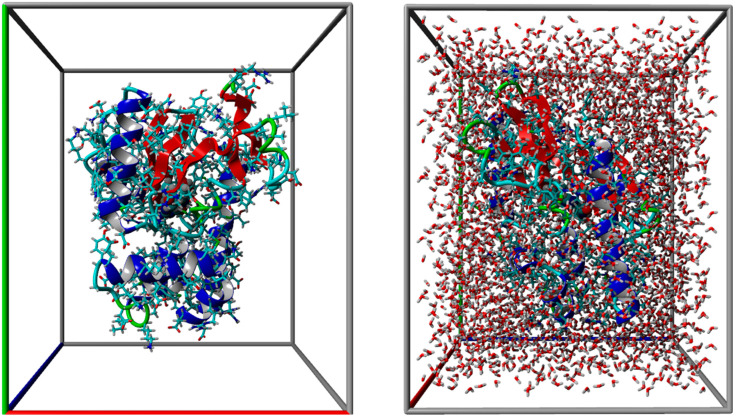
A ray-traced picture of the simulated system (snapshot). The simulation cell boundary is set to periodic. Atoms that stick out of the simulation cell will be wrapped to the opposite side of the cell during the simulation.

To evaluate the stability of ligand binding under dynamic conditions, the poultry PA–entecavir complex was subjected to a 170 ns molecular dynamics simulation under explicit solvent conditions (Figure 4). Several trajectory-derived metrics were analyzed to assess structural stability.

**Figure 5:**
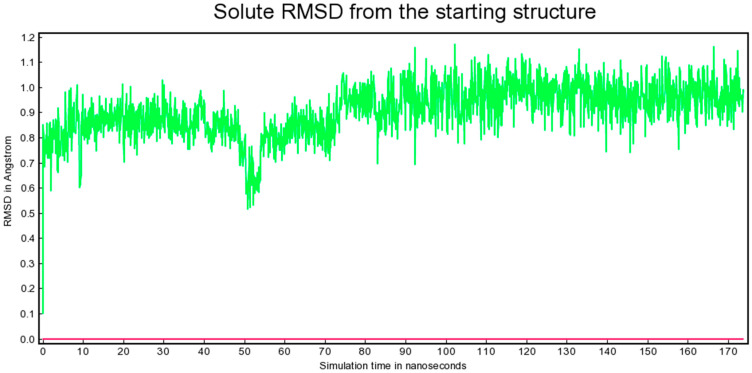
Solute RMSD from the starting structure for the poultry PA–entecavir complex over 170 ns YASARA MD simulation. RMSD stabilization indicates convergence and structural stability of the complex under explicit solvent conditions.

The root mean-square deviation (RMSD) of the solute relative to the starting structure indicated rapid equilibration early in the trajectory, followed by stable fluctuations below approximately 1.1 Å throughout the remainder of the simulation (Figure 5). This low RMSD range suggests that the protein–ligand complex maintained a stable conformational state over the simulation timescale.

**Figure 6:**
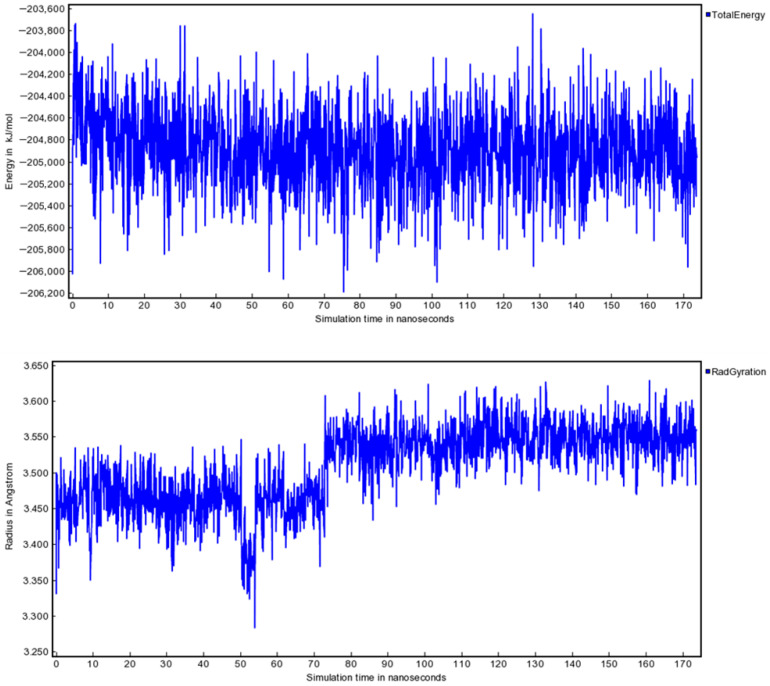
Total potential energy profile of the poultry PA–entecavir simulation system over 170 ns. Fluctuations occur around a stable mean without systematic drift, supporting stable simulation behavior. RMSD stabilization indicates convergence and structural stability of the complex under explicit solvent conditions.

The total potential energy profile fluctuated around a stable mean value without systematic drift (Figure 6), indicating that the system remained thermodynamically stable under the selected simulation conditions. Hydrogen bonding interactions between entecavir and key binding site residues remained stable throughout the trajectory, supporting sustained ligand engagement.

Analysis of the secondary structure content during the simulation revealed no major changes in helix or β-sheet fractions, confirming that ligand binding did not induce destabilisation of the PA endonuclease fold (Figure S1).

Residue-level RMSF analysis showed that most residues exhibited low fluctuation values, consistent with a structurally stable protein. Higher fluctuations were restricted primarily to solvent-exposed loop regions, a behavior typical for flexible surface segments of globular proteins (Figure S2).

Binding free energy estimation using the MM/PBSA approach yielded a mean ΔG_binding of −85.146 ± 0.836 kJ/mol, indicating energetically favorable complex formation and further supporting the stability of the entecavir-bound state throughout the simulation trajectory. The magnitude of the calculated binding free energy is consistent with stable protein–ligand complex formation and supports the docking results, indicating favorable interaction of entecavir with the avian PA catalytic site.

### 3.5. ADME Profiling and Selection Rationale

Physicochemical profiling performed using SwissADME (https://www.swissadme.ch) accessed on 4 March 2026, highlighted notable differences between the two candidate compounds (Table 6).

Entecavir exhibited a lower molecular weight (277.28 g/mol) compared with baloxavir (483.49 g/mol), along with a substantially higher polar surface area (TPSA 130.05 Å^2^) and markedly lower lipophilicity (consensus LogP −0.54). These characteristics correspond to a high predicted aqueous solubility, which may be advantageous for applications involving water-based delivery systems, such as drinking water treatments or environmental formulations.

Baloxavir displayed moderate lipophilicity (LogP ≈ 2.9) and lower polarity (TPSA 100.31 Å^2^), characteristics consistent with its design as a systemically administered antiviral drug. Both molecules showed no PAINS alerts, suggesting a low likelihood of assay interference.

Predicted pharmacokinetic properties indicated high gastrointestinal absorption for both compounds. However, baloxavir demonstrated predicted inhibition of several CYP isoforms (CYP2C19, CYP2C9, and CYP2D6). In contrast, entecavir showed no predicted CYP inhibition, suggesting a potentially more favorable metabolic interaction profile for drug repurposing.

### 3.6. Concise Endocrine Nuclear Receptor Screening

To explore potential off-target interactions relevant to toxicological safety, both compounds were screened against human nuclear receptors using the Endocrine Disruptome platform (Table S3).

Entecavir demonstrated moderate predicted docking affinities across several receptors, including the androgen receptor (AR), estrogen receptor α (ERα), mineralocorticoid receptor (MR), and thyroid receptor β (TRβ), with docking scores typically ranging from −7 to −8 kcal/mol equivalents.

Baloxavir generally showed weaker interactions across most receptors, although moderate docking scores were observed in antagonist-mode predictions for ERα, GR, and RXRα.

It should be noted that these predictions represent docking-based affinity estimates rather than functional endocrine activity. Still, they provide a preliminary toxicological context for evaluating candidate compounds within a broader One Health drug repurposing pipeline.

## 4. Discussion

This study presents a structured computational framework for the identification and prioritization of influenza A PA endonuclease inhibitors aligned with a One Health perspective. Cross-host structural modeling, molecular docking, molecular dynamics simulations, and physicochemical filtering were integrated into the workflow to move beyond conventional affinity-driven screening and incorporate parameters relevant to potential use in poultry-associated environments. Importantly, the present work should be interpreted as a computational prioritization rather than a definitive validation of antiviral efficacy.

The PA endonuclease remains a well-established antiviral target due to its essential role in cap-snatching during viral transcription and its high degree of structural conservation across influenza A viruses. While previous computational studies have explored this target extensively, most have focused on human influenza strains or historical H5N1 variants [32,33]. In contrast, the present study specifically examines contemporary clade 2.3.4.4b variants, which currently dominate global outbreaks and are expanding their host range. Structural comparison of poultry-associated and mammalian-associated models confirmed conservation of the catalytic architecture, particularly the metal-coordinating residues (H41, E80, D108, E119), while also revealing local variations in pocket geometry that may influence ligand binding. These findings support the use of cross-host modeling as a relevant component of antiviral prioritization, although they do not imply functional equivalence across host systems [34,35].

Docking analysis identified baloxavir and entecavir as compounds of interest within the screened library. As expected, baloxavir, a clinically validated PA endonuclease inhibitor, demonstrated consistent engagement with catalytic residues and served as an appropriate positive control across all targets. Entecavir, in contrast, is a nucleoside analog originally developed for the treatment of hepatitis B virus infection and does not belong to the classical metal-chelating scaffold typical of PA endonuclease inhibitors. The favorable docking behavior observed for entecavir, particularly within the poultry-associated model, suggests that it may adopt a stabilizing configuration within the catalytic pocket under the present modeling conditions. Although entecavir does not surpass baloxavir in the crystal structure or mammalian model, its improved docking score in the poultry-associated model (−100.6 vs. −97.5 to −97.7) suggests a target-dependent binding preference that warrants further investigation. However, this interaction should be interpreted cautiously, as it may reflect a non-canonical binding mode rather than the established metal-chelation mechanism observed for compounds such as baloxavir. Accordingly, entecavir is best described as a computationally prioritized candidate for further investigation, rather than a confirmed PA inhibitor [36].

Molecular dynamics simulations provided additional insight into the stability of the poultry PA–entecavir complex. The observed RMSD stability (<1.1 Å), consistent potential energy profile, and preservation of secondary structure suggest that the complex remains structurally stable over the simulated timescale. The calculated binding free energy (ΔG ≈ −85 kJ/mol) further supports an energetically favorable interaction under the simulation conditions. Nevertheless, it is important to emphasize that MD simulations were applied here as a focused validation step for the top-ranked candidate, and the purpose was not a comprehensive comparative analysis across multiple ligands or host systems. As such, these findings support internal consistency of the computational pipeline.

A distinguishing feature of this study is the incorporation of physicochemical and ADMET considerations relevant to poultry production environments. Conventional antiviral discovery pipelines typically prioritize compounds based on binding affinity and pharmacokinetic properties tailored for human systemic administration. In contrast, the present workflow includes filters for solubility, lipophilicity, and predicted metabolic interactions, which may be relevant for non-systemic or environmental applications. In this context, entecavir exhibited characteristics such as high polarity, low lipophilicity, and predicted aqueous solubility, which may suggest compatibility with water-based delivery concepts. Such considerations remain theoretical at this stage, and no conclusions regarding practical deployment or environmental application can be drawn without experimental validation.

The concept of integrating chemical suitability with antiviral target engagement reflects a broader shift toward translationally informed computational screening. With the combination of structural modeling with basic physicochemical filters, the workflow aims to prioritize compounds that are not only mechanistically plausible but also aligned with real-world constraints. This approach is particularly relevant in the context of H5N1 outbreaks, where interventions must operate across the animal–environment–human interface.

Several considerations need clarification here: first, the study is entirely computational and does not include biochemical or virological validation. Docking was performed using a single engine (iGEMDOCK), and although internal consistency was assessed through repeated runs, consensus docking approaches were not employed. The results were evaluated using molecular dynamics, a justified approach in computational studies. Second, molecular dynamics simulations were limited to a single ligand–target system, and comparative simulations with reference inhibitors or alternative host models were not conducted, since the poultry model is the one of interest here. Third, the compound library, while intentionally designed to reflect translational considerations, is limited in size and chemical diversity and does not encompass the entire chemical space, despite FDA-approved drugs also being screened here. Fourth, the Tier classification system for environmental or poultry suitability is conceptual and based on predictive parameters.

Future work should focus on validating the computational predictions presented here through experimental approaches. These include biochemical PA endonuclease inhibition assays and antiviral testing in avian-derived cell systems. Investigation of compound behavior in biologically relevant matrices and assessment of stability under poultry production conditions would also be necessary to support any translational development.

## 5. Conclusions

This study presents a structured computational framework for the identification and prioritization of influenza A PA endonuclease inhibitors within a One Health context. By integrating cross-host homology modeling, molecular docking, molecular dynamics simulations, and physicochemical filtering, the workflow enables the selection of candidate compounds based on both structural compatibility and basic translational considerations.

Within this framework, entecavir emerged as a computationally prioritized candidate, demonstrating favorable binding behavior in the poultry-associated PA model and stable interaction during molecular dynamics simulations. In addition, its predicted physicochemical profile contributed to its prioritization within the present computational workflow. However, these findings do not establish inhibitory activity, antiviral efficacy, or suitability for environmental or poultry applications.

The primary contribution of this work lies in the development of an integrative prioritization strategy that extends beyond conventional affinity-based screening by incorporating cross-host structural evaluation and basic suitability filters relevant to real-world contexts. The results provide a basis for future experimental validation, including biochemical PA inhibition assays, avian cell-based studies, and further computational refinement.

Overall, this framework offers a scalable approach for early-stage antiviral candidate prioritization against contemporary H5N1 variants and supports the integration of computational drug discovery with broader One Health considerations.

## Figures and Tables

**Table 1:** Ligand library used for initial screening against H5N1 PA endonuclease Group A—Reference PA endonuclease inhibitors (protocol validation, *n* = 10).

ID	Compound Name	Chemical Class/Role	Rationale
A1	Baloxavir (baloxavir acid)	Approved PA endonuclease inhibitor	Gold-standard positive control
A2	Baloxavir marboxil	Prodrug of baloxavir	Comparator; shows prodrug vs. active
A3	L-742,001	Experimental PA inhibitor	Widely cited research inhibitor
A4	2,4-Dioxo-4-phenylbutanoic acid	2,4-Dioxobutanoic acid	Classic PA metal-chelating scaffold
A5	4-(4-Chlorophenyl)-2,4-dioxobutanoic acid	2,4-Dioxobutanoic acid	Aryl-substituted PA inhibitor
A6	4-(4-Fluorophenyl)-2,4-dioxobutanoic acid	2,4-Dioxobutanoic acid	Aryl-substituted PA inhibitor
A7	4-(4-Bromophenyl)-2,4-dioxobutanoic acid	2,4-Dioxobutanoic acid	Aryl-substituted PA inhibitor
A8	3-Hydroxyquinolin-2(1H)-one	Hydroxyquinolinone	PA inhibitor pharmacophore
A9	3-Hydroxypyridin-2(1H)-one	Hydroxypyridinone	PA inhibitor pharmacophore
A10	Flutimide	Historic PA-inhibitor scaffold	Literature comparator

**Table 2:** Ligand library used for initial screening against H5N1 PA endonuclease Group B—Hydrophilic, metal-binding “poultry-friendly” candidates (*n* = 20).

ID	Compound Name	Chemical Class	Rationale
B1	Gallic acid	Polyphenolic acid	Strong metal chelation, food-adjacent
B2	Caffeic acid	Phenolic acid	Metal binding, antioxidant
B3	Ferulic acid	Phenolic acid	Hydrophilic, feed-relevant
B4	p-Coumaric acid	Phenolic acid	Small, polar aromatic acid
B5	Protocatechuic acid	Dihydroxybenzoic acid	Catechol-type chelator
B6	Gentisic acid	Dihydroxybenzoic acid	Metal binding, polar
B7	Chlorogenic acid	Polyphenol	Potent chelator, larger scaffold
B8	Catechol	Simple diol	Minimal chelation motif
B9	Pyrogallol	Trihydroxybenzene	Strong chelation motif
B10	Salicylic acid	Hydroxybenzoic acid	Classic chelating pharmacophore
B11	Acetohydroxamic acid	Hydroxamate	Strong metalloenzyme binder
B12	Benzohydroxamic acid	Hydroxamate	Drug-like chelator
B13	Deferiprone	Hydroxypyridinone	Potent metal chelator
B14	Maltol	Hydroxypyrone	Moderate chelator
B15	Kojic acid	Hydroxypyrone	Metal-binding scaffold
B16	Pyridine-2,4-dicarboxylic acid	Heteroaromatic diacid	PA-relevant chelation geometry
B17	Pyridine-2,6-dicarboxylic acid	Heteroaromatic diacid	Dipicolinic acid, a strong chelator
B18	Pyridine-3,5-dicarboxylic acid	Heteroaromatic diacid	Symmetric chelation
B19	Phthalic acid	Aromatic diacid	Compact diacid scaffold
B20	Isophthalic acid	Aromatic diacid	Positional isomer comparator

**Table 3:** Ligand library used for initial screening against H5N1 PA endonuclease Group C—Environmental/food system comparators (One Health context, *n* = 10).

ID	Compound Name	Chemical Class	Rationale
C1	Citric acid	Tricarboxylic acid	GRAS chelator
C2	Lactic acid	Organic acid	Food system relevance
C3	Malic acid	Dicarboxylic acid	GRAS, polar
C4	Tartaric acid	Dicarboxylic acid	GRAS, chelating
C5	Succinic acid	Dicarboxylic acid	Simple aliphatic diacid
C6	Fumaric acid	Dicarboxylic acid	Unsaturated diacid
C7	Gluconic acid	Polyhydroxy acid	Food and sanitation use
C8	Ascorbic acid	Vitamin C	Redox-active, chelating
C9	EDTA	Polyaminocarboxylate	Strong metal chelator (reference)
C10	Phytic acid	Polyphosphate	Strong chelator, feed relevance

**Table 4:** Molecular dynamics summary for poultry PA–entecavir.

Metric	Observation
Simulation length	170 ns
RMSD (solute vs. start)	Stabilizes during trajectory; plateau below ~1.1 Å
Total potential energy	Fluctuates around stable mean; no systematic drift
RMSF	Expected local flexibility; no global destabilization indicated
Secondary structure	Helix/sheet content remains broadly stable

**Table 5:** iGEMDOCK docking energies (total score) across three PA targets.

Target	Ligand	Docking Energy (Reported)	Notes
6FS8 (crystal)	Baloxavir	−101.7, −101.7, −101.6	replicate runs consistent
6FS8 (crystal)	Entecavir	−83.0, −83.0	replicate runs consistent
Poultry PA model	Baloxavir	−97.5, −97.7	replicate runs
Poultry PA model	Entecavir	−100.6, −100.6	replicate runs
Mammalian (fox) PA model	Baloxavir	−97.6 to −97.7	replicate runs
Mammalian (fox) PA model	Entecavir	−95.0 to −95.1	replicate runs

**Table 6:** SwissADME summary.

Parameter	Entecavir	Baloxavir (Acid)
Molecular weight (g/mol)	277.28	483.49
TPSA (Å^2^)	130.05	100.31
Consensus LogP	−0.54	2.90
HBA/HBD	5/4	6/1
GI absorption	High	High
BBB permeant	No	No
P-gp substrate	No	No
PAINS	0 alerts	0 alerts
CYP inhibition (selected)	None predicted	CYP2C19/CYP2C9/CYP2D6 Predicted
Solubility class	Soluble/very soluble	Moderately soluble

## Data Availability

The data presented in this study are available on request from the corresponding author. The data are not publicly available due to practical limitations.

## Supplementary Materials

The following supporting information can be downloaded at: https://www.mdpi.com/article/10.3390/vetsci13040385/s1, Figure S1: Protein secondary structure content (helix, sheet, turn, coil) across the 170 ns poultry PA–entecavir trajectory. The stability of secondary structure fractions indicates preserved fold integrity during MD; Figure S2: Root mean square fluctuation (RMSF) profile of the poultry influenza A PA–entecavir complex obtained from the 170 ns molecular dynamics simulation performed using YASARA Dynamics. RMSF values were calculated for each residue to evaluate local structural flexibility during the trajectory. Most residues exhibit low fluctuation values, indicating overall structural stability of the protein–ligand complex, while slightly higher fluctuations correspond to the solvent-exposed loop region; Table S1a: Group A—Reference PA endonuclease inhibitors (SMILES); Table S1b: Group B—Hydrophilic, metal-binding candidates (SMILES); Table S1c: Group C—Environmental/food system comparators (SMILES); Table S2: Overview of targets, modeling, and computational workflow; Table S3: Endocrine Disruptome nuclear receptor docking-based screening (entecavir vs. baloxavir).
