# Supplementary material for: A One Health Computational Framework for Identifying PA Endonuclease Inhibitors Against Contemporary H5N1 Avian Influenza
Source: Vet Sci. 2026 Apr 16;13(4):385. doi: 10.3390/vetsci13040385 (PMC13120429; doi:10.3390/vetsci13040385)
Supplement: Supplementary file 1 [file vetsci-13-00385-s001.zip › vetsci-4212718-supplementary.pdf]

Supplementary Material - vetsci-4212718

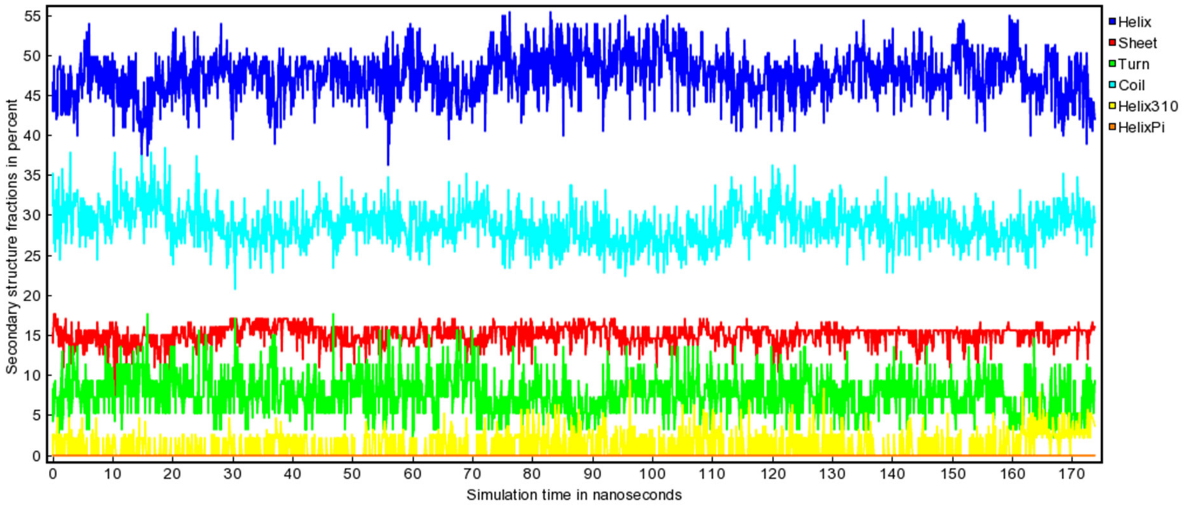

Figure S1. Protein secondary structure content (helix, sheet, turn, coil) across the 170 ns poultry PA–entecavir trajectory. The stability of secondary structure fractions indicates preserved fold integrity during MD.

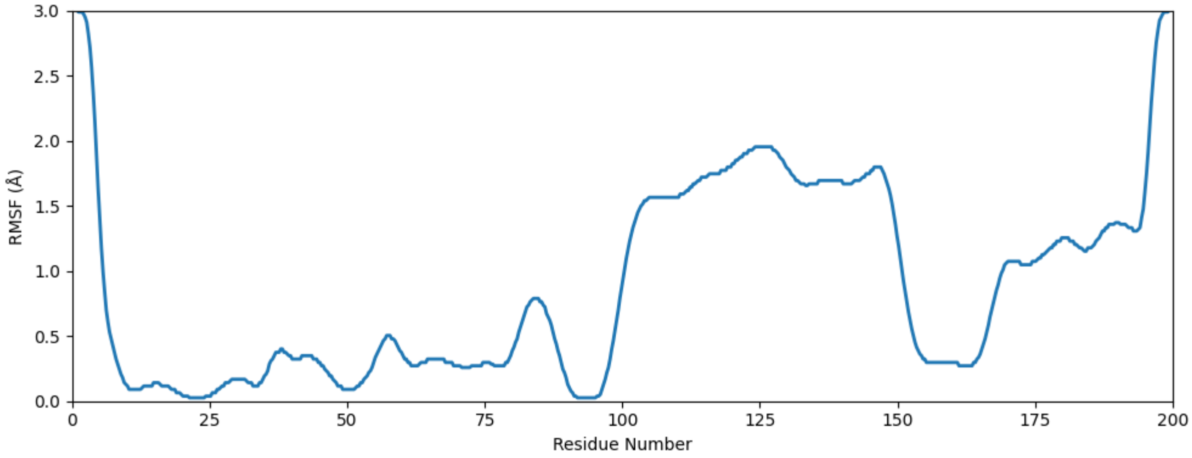

Figure S2. Root mean square fluctuation (RMSF) profile of the poultry influenza A PA–entecavir complex obtained from the 170 ns molecular dynamics simulation performed using YASARA Dynamics. RMSF values were calculated for each residue to evaluate local structural flexibility during the trajectory. Most residues exhibit low fluctuation values, indicating overall structural stability of the protein–ligand complex, while slightly higher fluctuations correspond to solvent-exposed loop region.

Table S1a. Group A – Reference PA endonuclease inhibitors (SMILES)

ID	Compound	Canonical SMILES
A1	Baloxavir (baloxavir acid)	C1COC[C@@H]2N1C(=O)C3=C(C(=O)C=CN3N2[C@H]4C5=C(CSC6=CC=CC=C46)C(=C(C=C5)F)F)O
A2	Baloxavir marboxil	COC(=O)OCOC1=C2C(=O)N3CCOC[C@H]3N(N2C=CC1=O)[C@H]4C5=C(CSC6=CC=CC=C46)C(=C(C=C5)F)F
A3	L-742,001	Cl.OC(=O)C(O)=CC(=O)C1(Cc2ccc(Cl)cc2)CCN(Cc2ccccc2)CC1
A4	2,4-Dioxo-4-phenylbutanoic acid	O=C(O)C(C(=O)C(=O)c1ccccc1)
A5	4-(4-Chlorophenyl)-2,4-dioxobutanoic acid	O=C(O)C(C(=O)C(=O)c1ccc(Cl)cc1)
A6	4-(4-Fluorophenyl)-2,4-dioxobutanoic acid	O=C(O)C(C(=O)C(=O)c1ccc(F)cc1)
A7	4-(4-Bromophenyl)-2,4-dioxobutanoic acid	O=C(O)C(C(=O)C(=O)c1ccc(Br)cc1)
A8	3-Hydroxyquinolin-2(1H)-one	O=c1nc(O)c2ccccc2[nH]1
A9	3-Hydroxypyridin-2(1H)-one	O=c1nc(O)cc[nH]1
A10	Flutimide	O=C1NC(=O)N(CC1)c2ccc(F)cc2

Table S1b. Group B – Hydrophilic, metal-binding candidates (SMILES)

ID	Compound	SMILES
B1	Gallic acid	O=C(O)c1cc(O)c(O)c(O)c1
B2	Caffeic acid	O=C(O)/C=C/c1ccc(O)c(O)c1
B3	Ferulic acid	COc1cc(/C=C/C(=O)O)ccc1O
B4	p-Coumaric acid	O=C(O)/C=C/c1ccc(O)cc1
B5	Protocatechuic acid	O=C(O)c1cc(O)c(O)cc1
B6	Gentisic acid	O=C(O)c1cc(O)cc(O)c1
B7	Chlorogenic acid	O=C(O)[C@@H](O)C(O)c1ccc(O)c(O)c1
B8	Catechol	Oc1ccccc1O
B9	Pyrogallol	Oc1cc(O)c(O)cc1
B10	Salicylic acid	O=C(O)c1ccccc1O
B11	Acetohydroxamic acid	CC(=O)NO
B12	Benzohydroxamic acid	O=C(NO)c1ccccc1
B13	Deferiprone	CC1=CC(=O)NC(=O)C1
B14	Maltol	CC1=CC(=O)OC(O)=C1
B15	Kojic acid	OC1=COC(=O)C(O)=C1
B16	Pyridine-2,4-dicarboxylic acid	O=C(O)c1cc(nc(C(=O)O))cc1
B17	Pyridine-2,6-dicarboxylic acid	O=C(O)c1nc(C(=O)O)ccc1
B18	Pyridine-3,5-dicarboxylic acid	O=C(O)c1cc(C(=O)O)nc(C(=O)O)c1
B19	Phthalic acid	O=C(O)c1ccccc1C(=O)O
B20	Isophthalic acid	O=C(O)c1ccc(cc1)C(=O)O

Table S1c. Group C – Environmental / food-system comparators (SMILES)

ID	Compound	SMILES
C1	Citric acid	O=C(O)C(CC(=O)O)(O)C(=O)O
C2	Lactic acid	CC(O)C(=O)O
C3	Malic acid	O=C(O)CC(O)C(=O)O
C4	Tartaric acid	O=C(O)[C@H](O)[C@H](O)C(=O)O
C5	Succinic acid	O=C(O)CCC(=O)O
C6	Fumaric acid	O=C(O)/C=C/C(=O)O
C7	Gluconic acid	O=C(O)C(O)C(O)C(O)C(O)CO
C8	Ascorbic acid	OC[C@H]1OC(O)=C(O)C(=O)C1O
C9	EDTA	O=C(O)CN(CCN(CC(=O)O)CC(=O)O)CC(=O)O
C10	Phytic acid	O=P(O)(O)O[C@H]1[C@H](OP(O)(O)=O)[C@H](OP(O)(O)=O)[C@H](OP(O)(O)=O)[C@H](OP(O)(O)=O)[C@H]1OP(O)(O)=O

Table S2. Overview of targets, modelling, and computational workflow

Item	Description	Output used in this study
Crystal target	Influenza A PA endonuclease template	PDB 6FS8
Homology model 1	Poultry-associated PA endonuclease (residues 1–193), SWISS-MODEL using 6FS8	Docking target + MD target
Homology model 2	Mammalian-associated (fox) PA endonuclease (residues 1–193), SWISS-MODEL using 6FS8	Docking target
Docking engine	iGEMDOCK	Energies + interaction residues
MD engine	YASARA Dynamics (GUI)	170 ns poultry PA–entecavir
Binding energy	MM/PBSA on equilibrated frames	ΔG = −85.146 ± 0.836 kJ/mol
ADME	SwissADME	Physchem + drug-likeness
Toxicology screen	Endocrine Disruptome	Nuclear receptor docking-based screening

Table S3. Endocrine Disruptome nuclear receptor docking-based screening (Entecavir vs Baloxavir)

Receptor	Entecavir	Baloxavir
AR	−7.7	4.4
AR antagonist	−8.3	0.2
ERα	−8.0	−5.6
ERα antagonist	−8.0	−8.1
ERβ	−7.7	0.7
ERβ antagonist	−7.6	−6.5
GR	−7.5	−4.5
GR antagonist	−7.0	−7.8
MR	−8.3	1.0
LXRα	−8.0	−4.7
LXRβ	−7.9	−3.9
PPARα	−7.8	−4.2
PPARβ	−8.0	−4.9
PPARγ	−6.7	−6.5
PR	−3.0	−3.0
RXRα	−7.6	−7.1
TRα	−7.6	4.1
TRβ	−8.0	3.2
